# Prebiotic effects of alfalfa (*Medicago sativa*) fiber on cecal bacterial composition, short-chain fatty acids, and diarrhea incidence in weaning piglets

**DOI:** 10.1039/c9ra01251f

**Published:** 2019-05-02

**Authors:** Seidu Adams, Kong Xiangjie, Jiang Hailong, Qin Guixin, Fredrick Leo Sossah, Che Dongsheng

**Affiliations:** College of Animal Science and Technology, Jilin Agricultural University Changchun 130118 China hljiang@jlau.edu.cn chedongsheng@jlau.edu.cn; Jilin Provincial Key Lab of Animal Nutrition and Feed Science, Jilin Agricultural University Changchun 130118 China; Key Lab of Animal Production, Product Quality and Security, Ministry of Education, Jilin Agricultural University Changchun 130118 China; College of Agronomy and Molecular Biology China

## Abstract

Dietary alfalfa fiber (AF) is conceived to modulate gut microbial richness and diversity to improve the health and growth of weaning piglets. The objective of this study was to evaluate the prebiotic effects of AF on diarrhea incidence, the production of short-chain fatty acids (SCFAs), and microbiota composition in weaning piglets. This study utilized 100 crossbred piglets (Duroc × Landrace × Yorkshire) with a body weight of 8.42 ± 1.88 kg randomly assigned to the following treatments: 0.00% AF meal (A), 6.00% of AF meal (B), 12.00% AF meal (C), and 18.00% AF meal (D). The cecum samples were used to determine microbial community composition and diversity through high-throughput 16S rDNA sequencing. The results of this study show that the lowest average daily gain (ADG) was observed in treatment D, and the highest ADG was recorded in treatment C. However there was no significant difference between the treatment groups and the control. The average daily feed intake (ADFI) was significantly higher in treatment C compared to the other treatments. The feed conversion ratio was high in the control group compared to the AF treated groups. The highest diarrhea incidence was observed in treatment A and the lowest diarrhea incidence was observed in treatment C and D. The highest acetate and propionate levels were observed in treatment B, but there was no significant difference between the treatment groups and the control. The supplementation of AF significantly increased the butyrate level in treatment D compared with treatments A and B but was not significantly different from treatment C. The Observed_species richness and Simpson diversity values of the cecum bacterial composition in the AF fed piglets were higher than the control. In addition, the Chao 1 richness and Shannon diversity increased with an increase in AF supplementation, reaching a plateau at treatment B and C, then decreasing at treatment D. The *Bacteroidetes*, *Firmicutes*, *Tenericutes*, *Proteobacteria*, *Cyanobacteria*, *Spirochaetae*, *Actinobacteria*, *Fibrobacteres*, *Saccharibacteria*, *Synergistetes*, *Chlamydiae*, *Elusimicrobia*, *Deferribacteres*, *Fusobacteria*, and others were relatively abundant in all treatments. The *Bacteroidetes* and *Firmicutes* were the dominant phyla, accounting for 98% of all reads. AF treatment decreased the *Bacteroidetes* phylum and increased the *Firmicutes* phylum compared with treatment A. Therefore, the dietary inclusion of AF may decrease diarrhea incidence, increase cecal bacterial composition and richness, and consequently improve the growth performance of weaning piglets.

## Abstract 2

Dietary alfalfa fiber (AF) is conceived to modulate gut microbial richness and diversity to improve the health and growth of weaning piglets.

## Graphical abstract

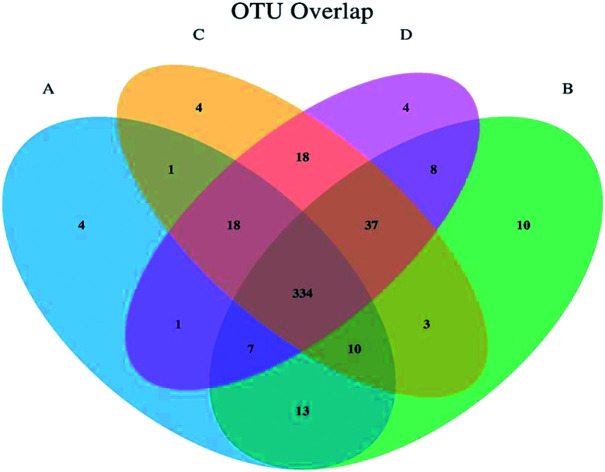

## 1 Introduction

The gut microbiota contains over 10^14^ microbial cells that collectively comprise at least 150 times more genes than their host.^1,2^ The composition of the gut microbiota in humans and other monogastric animals varies based on age, diet, genetic characteristics, and sex.^1^ Gut microbiota have some important functions in host health, nutrient metabolism and absorption, development of the host immune system, differentiation of the gut epithelium, and maintenance of the gut mucosal barrier functions.^3,4^ Previous evidence indicated that gut microorganisms can directly influence physiological conditions in animals such as improving the roles of the intestinal barrier, stimulating defence mechanisms against pathogens, improving the host immune system, increasing defensive mechanisms against inflammatory bowel diseases (IBD),^5^ regulating autoimmunity, producing biological metabolites, preventing obesity and regulating diabetes, and destroying cancer cells.^1,6^ The interactions between the host gut-microbiota are dynamic and highly vulnerable to several environmental conditions, particularly diet.^1^

Alfalfa is a high-yielding perennial legume that is cultivated worldwide with rich nutritional characteristics and bioactive compounds.^7,8^ Alfalfa fiber is mainly composed of insoluble dietary fiber, such as cellulose, lignin, and xylans, representing more than 90% of the total dietary fiber composition of alfalfa.^9,10^ Interestingly, alfalfa meal contains 17.5% CP, 24.1% CF, and 1200 kcal kg^−1^ metabolizable energy.^11^ Recent studies reported that pigs grown on alfalfa pastures produce good-quality natural pork, which is currently gaining more attention in Europe and North America.^12,13^ Interestingly, for the production of good quality pork, alfalfa is used as fresh fodder in some traditional style pig production systems in China^13^ and other parts of the world. AF is a high-quality protein feed; therefore, increasing its supplementation may improve the nutritional requirement of pigs and increase the growth of piglets. Few studies reported that the supplementation of natural compounds such as flavonoids extracted from alfalfa had no estrogenic impacts, which may increase the production performance, enhance antioxidants, induce anti-stress function, and eradicate free radicals from animals.^12,14^ Dietary fiber influence the role of the gut microbiota, as indicated by the low digestibility and availability of nutrients in feeds with high fiber levels.^1,15^ The type of dietary fiber, soluble or insoluble, influences its effects on gut microbial composition and function.^1,16,17^ The intake of high dietary fiber has been reported to influence energy metabolism, nutrient utilization, and subsequently results in a reduction in the performance of monogastric animals, especially pigs.^18–20^ Recent findings indicated that dietary fiber can prevent intestinal diseases such as constipation, diarrhea, bowel diseases, and improve intestinal health in both humans and animal subjects.^10,21,22^ Different dietary fiber has beneficial roles in gut microbial composition and function. Such beneficial roles include SCFAs synthesis, decreased intestinal pH, and fecal bulkiness.^1,21,23^ It was reported that dietary fiber may selectively modulate intestinal microbiota *via* the stimulation of constructive microbial species and the suppression of enteric bacterial species.^10,24^ Dietary fiber functions as a nutrient for intestinal microbiota and consequently motivates the growth of valuable intestinal bacteria, like *Bifidobacterium*, which are involved in the reduction in cardiovascular disorders.^25^

Weaning piglets are associated with a reduction in feed consumption and low nutrient metabolism due to the immature digestive systems, stress as a result of the separation from the sows and litter mates, and the sudden change from the easily digestible sow's milk to less digestible solid feed.^12,16,17,26^ The treatment of high-fiber diets to piglets results in an overall increase in the total empty-weight of the gut^27^ and an increase in gut secretions.^28^ Dietary fiber increased the growth performance, nutrient digestibility, and immunity in piglets,^29,30^ resulting in a decrease in diarrhea incidence.^31,32^ There was a report that dietary fiber supplementation may improve growth performance and decrease the intestinal permeability in piglets,^31,33^ indicated that dietary fiber extracted from *Astragalus membranaceus* increased growth performance, nutrient digestibility, VFA production, and immunity in piglets. However, the effects of AF on gut microbiota composition, health, and energy metabolism in piglets are limited.

Therefore, the objective of this study was to evaluate the prebiotic effects of AF on diarrhea incidence, SCFA production, and microbiota composition in weaning piglets. This objective was investigated by feeding different concentrations of AF to 28 day-old piglets for 48 days and the cecal samples were extracted for microbial analysis. The 16S rDNA pyrosequencing technique was used to examine the prebiotic effects of AF on the cecal microbial composition, and the growth parameters of the piglets were examined.

## 2 Materials and methods

### 2.1 Animal care and ethics

All animal procedures were carried out in accordance with the Guidelines for the Care and Use of Laboratory Animals of Jilin Agricultural University, and experiments were approved by the Animal Ethics Committee of Jilin Agricultural University.

### 2.2 Experimental animals, diet, and sampling

A total of 100 crossbred piglets (Duroc × Landrace × Yorkshire) weaned at 28 days of age with a mean body weight of 8.42 ± 1.88 kg were randomly allocated to 4 treatment groups with 5 replicates per treatment and 5 pigs per replicate using the randomized complete block design. The piglets were housed in pens (1.8 × 1.2 m) with a concrete slatted floor and *ad libitum* access to feed and water. The room temperature was maintained at 26 °C at the time of weaning and progressively reduced to 22 °C within the first week of post weaning. The humidity was kept constant at 65–75%. The piglets in each group received one of the 4 experimental diets (Table 1) with cracked corn and soybean meal as the main source of energy and protein, respectively. The diets were formulated as follows:^34^ the control diet of 0.00% AF (A), 6.00% AF diet (B), 12.00% AF diet (C), and 18.00% AF diet (D). All nutritional components in Table 1 were supplement based on the national nutrient requirements of piglets with the exceptions of AF supplementation. Therefore, the obtained results were analysed based on the added AF levels. The experiment lasted for 48 days and 7 days of pre-feeding tests. There was no antibiotic supplementation before and during the experimental trial. Piglets were weighed individually at the beginning and the end of the experiment and daily feed intake per pen was recorded. At the end of the feeding trial, 10 pigs were randomly selected from each group. After fasting overnight, the pigs were slaughtered, and the gastrointestinal tract was immediately removed. The cecal content was collected into 5 mL cryotubes and snap-frozen immediately in liquid nitrogen and stored at −80 °C until further microbial and metagenomics analysis. In addition, 50 mL centrifuge tube of cecum digest samples were collected and frozen in −20 °C for SCFAs analysis.

**Table 1:** Shows the composition and nutritional levels of the basal diets (%)^a^

	A	B	C	D
**Items**				
Corn	53.50	50.29	45.00	40.00
Soybean meal	28.00	26.32	24.64	22.00
AF	0.00	6.00	12.00	18.00
Fish meal	3.00	2.82	3.69	5.10
Whey powder	9.00	5.50	5.50	5.00
Wheat bran	1.00	1.00	1.00	1.00
Limestone powder	1.30	1.22	1.14	0.90
Calcium hydroxide	0.80	0.75	0.70	0.66
Wheat middling feed	2.00	1.88	1.76	1.64
Rapeseed oil	0.60	1.50	3.56	4.70
Premix	1.00	1.00	1.00	1.00

**Nutritional level**				
Digestive energy Mcal kg^−1^	3.41	3.33	3.31	3.26
Crude protein%	20.10	20.02	20.06	20.27
Crude fiber%	2.83	4.21	5.55	6.84
Lysine%	1.14	1.12	1.12	1.13
Methionine% + cysteine%	0.69	0.67	0.65	0.65
Threonine%	0.81	0.81	0.81	0.82
Isoleucine%	0.87	0.86	0.86	0.87
Leucine%	1.72	1.68	1.65	1.64
Proline%	0.92	0.92	0.92	0.91
Phenylalanine%	0.99	0.98	0.98	0.99
Tryptophan%	0.30	0.32	0.34	0.38
Calcium to phosphorus ratio	2 : 1	2 : 1	2 : 1	2 : 1

a Note: premix is available per kg of feed: VA 130–396 KIU, VD2 30–124 KIU, VE 400 mg, VK2 40–150 mg, VB1 25 mg, VB2 75 mg, Cu 1500–7500 mg, Fe 1500–7500 kg, Zn 1500–3700 kg, Mn 400–3700 kg, moisture 9%, sodium chloride 6–14%, total phosphorus 2%, lysine 1.3%, calcium 10–20%, phytase 12 500 U. A: 0.00% AF, B: 6.00% AF, C: 12.00% AF, D: 18.00% AF.

### 2.3 Growth performance, diarrhea incidence, and SCFAs

The initial body weight of the piglets was subtracted from the final body weight and divided by the days of the experiment to obtain the ADG. The ADFI was calculated by estimating the total feed intake per day. The FCR was calculated by dividing the feed intake by the body weight gain. All piglets were checked for the consistency in fecal samples as an indicator of their health status. The fecal samples were recorded as normal, pasty, or watery.^35^ The diarrhea incidence (%) was calculated using the method.^36^ The acetate, butyrate, propionate, and the total SCFAs were analyzed according to the method described.^37^

### 2.4 Total DNA extraction

Total DNA was extracted and quantified from 0.5 g of each cecal samples using a Qubit 2.0 Fluorometer (Thermo Fisher Scientific Inc. Massachusetts, USA) following the manufacturer's instructions. The amount of total DNA was estimated by the NanoPhotometer spectrophotometer (Thermo Fisher Scientific Inc. Massachusetts, USA), and the molecular size and DNA quality were measured using 1% agarose gel electrophoresis. Cecal sample preparation was performed as described.^31^ All DNA samples were stored at −20 °C until further analysis.

### 2.5 16S rDNA gene amplification and sequencing

Sequencing was performed at the Annotech Genentech (Beijing Co., Ltd). Briefly, 10 ng of DNA template was used to amplify the target region, according to the different sequencing regions, the corresponding primers: 341F (5′-CCTACACGACGCTCTTCCGATCTN-3′) and 805R (5′-GACTGGAGTTCCTTGGCACCCGAGAATTCCA-3′) using TaKaRa's Ex Taq enzyme to ensure amplification efficiency and accuracy. The preliminary quantification was performed using Qubit 2.0. Then, the insert size of the library was detected using Agilent 2100. The Bio-Rad CFX 96 real-time PCR instrument was used to perform the qPCR to accurately quantify the effective concentration of the library to ensure library quality. The PCR was performed as described.^31^ Then, the mixture of the PCR product was purified with a GeneJET Gel Extraction Kit (Thermo Fisher Scientific). The sequencing libraries were generated using a NEB Next Ultra DNA Library Prep Kit for Illumina (NEB, United States) following the manufacturer's instructions. The library quality was assessed on the Qubit@2.0 Fluorometer (Life Technologies, CA, United States) and the Agilent Bioanalyzer 2100 system. Finally, the library was sequenced on an Illumina HiSeq platform, and paired-end reads were generated.

### 2.6 Sequence analysis

For the sequences obtained by the sequencing, data filtering was completed by removing the low-quality base, Ns and joint contamination sequences to obtain a reliable target sequence for subsequent analysis. The filtered sequences were referred to as the Clean Reads. First, the corresponding Read1 and Read2 of the sequence fragments were obtained from the 5′ and 3′ ends, respectively using the sequence stitching method PEAR;^38^ the spliced sequence was analyzed using the QIIME software version 1.80,^39–41^ including the extraction of operational taxonomic units (OTUs), overlap analysis, cluster analysis, Lefse analysis, phylogenetic tree construction, alpha diversity analysis, beta diversity analysis, *etc.*^42^

The operational taxonomic units were clustered with a 97% similarity threshold. Species identification was based on the comparison of the corresponding recognition sequences of each species in the database with the OTUs. Operational taxonomic units with fewer than 2 sequences in all samples were eliminated to rule out the impact of less reliable OTUs on subsequent analysis. Change in OTUs between samples was counted and represented in heat maps. The number of unique OTUs for each group was represented on a Venn diagram. The distribution of species in each sample at each classification level was represented in histograms. According to the species annotation and abundance information of all the samples at the class, family, and genus levels, the top 25 subjects were selected based on the families, genera, and their abundance information in each sample to draw sample heat maps, and classify them according to the classification standards. Sample clustering was perform using the information available at each sample level for the easy display of results and the discovery of information to identify more species or samples that were concentrated in the sample study for the obtained species distribution data of each sample after normalization. The Bray–Curtis distance value was calculated, and each sample was clustered by a hierarchical clustering algorithm. The cluster analyzed the similarities between samples.

According to the species content of the samples, the distribution results at the five taxonomic levels comprised of the phylum, class, order, family, and genus were calculated. The information available at the phylum, class, order, family, and genus levels were used to draw species distribution histograms. For the identification of the dominant species in each sample, the dominance index for each species in each sample was calculated. For species similarities and diversity, the alpha diversity analysis using the Shannon diversity index, Simpson diversity, Chao 1 richness, and observed species richness were used. The principal component analysis (PCA) was performed at the genus level,^43^ and the results were visualized using the Statistical Analysis of Metagenomics Profiles (STAMP) version 2.13 program.^44^ The linear discriminant analysis coupled with effect size (LEfSe) was performed to identify the bacterial taxa differentially represented between groups at the genus or higher taxonomy levels.^45^ LefSe analysis combines linear discriminant analysis with the KW rank sum test and Wilcox test to determine the effect of different microbial species in different treatment groups. Finally, the Metastats software and T-test software were used to analyzed the species abundance data between groups.

### 2.7 Data analysis

Data were analyzed using a one-way analysis of variance (ANOVA) procedure of SPSS software version 20.0 (SPSS Inc., Chicago, IL, USA). A probability value of *p* < 0.05 was considered statistically significant and differences between means were noticeable. The Duncan Multiple Range Test (DMRT) was employed to ascertain the differences between the treatments.

## 3 Results

### 3.1 Growth performance and piglet diarrhea

The growth performance of piglets increased with the increase in AF supplementation, but at higher AF concentrations (D), there was a decrease in the growth performance of piglets. There was no significant difference (*p* > 0.05) in the ADG among all the treated groups. The ADFI of treatment C was significantly different (*p* < 0.05) from treatment A and treatment D but not significantly different (*p* > 0.05) from treatment B. Treatment D recorded the highest F : G with no significant difference (*p* > 0.05) between the treatments. The diarrhea incidence in treatment A was significantly higher (*p* < 0.05) than the other treatment groups. Meanwhile, the lowest diarrhea incidence was recorded in treatment C and treatment D (Table 2).

**Table 2:** Effects of AF on the growth performance and diarrhea incidence in weaned piglets (kg)

Items	A	B	C	D
ADG	1.16 ± 0.17^a^	1.25 ± 0.19^a^	1.32 ± 0.16^a^	1.04 ± 0.23^a^
ADFI	2.02 ± 0.55^b^	2.13 ± 0.22^a,b^	2.22 ± 0.19^a^	1.89 ± 0.06^b,c^
F : G	1.74 ± 0.20	1.70 ± 0.23	1.68 ± 0.38	1.82 ± 0.32
Diarrhea (%)	6.94 ± 0.12^a^	4.17 ± 0.25^b^	0.37 ± 0.21^c^	0.35 ± 0.11^c^

a Note: A: 0.00% AF, B: 6.00% AF, C: 12.00% AF, D: 18.00% AF. Different alphabets in the same column denote a significant difference, and the same alphabets or no alphabets means that they were not significantly different. A significant difference was observed at *p* < 0.05.

### 3.2 Cecal short-chain fatty acids production in pigs

As shown in Table 3, the supplementation of AF increased the acetate and propionate levels. The highest acetate and propionate levels were observed in treatment B, but there was no significant difference (*p* > 0.05) between the treatment groups. The butyrate concentration increased with the increase in AF concentration. There was a significant increase (*p* < 0.05) in butyrate production in treatments C and D in comparison with treatments A and B. The highest total SCFAs was observed in treatment B, but there was no significant difference (*p* > 0.05) between the groups.

**Table 3:** Effect of AF supplementation on short-chain fatty acids in the cecum of piglets (mmol L^−1^)

Items	A	B	C	D
Acetate	35.154 ± 5.93^a^	43.27 ± 8.97^a^	39.84 ± 2.11^a^	38.17 ± 5.82^a^
Propionate	29.92 ± 1.41^a^	32.78 ± 1.73^a^	31.96 ± 4.03^a^	31.72 ± 4.30^a^
Butyrate	21.56 ± 1.66^b^	22.13 ± 2.58^b^	25.21 ± 2.35^a^	25.78 ± 7.17^a^
Total SCFAs	86.63 ± 6.75	98.18 ± 10.01	97.01 ± 4.73	95.67 ± 3.18

a Note: A: 0.00% AF diet, B: 6.00% AF diet, C: 12.00% AF diet, D: 18.00% AF diet. Different alphabets in the same column denote a significant difference, and the same alphabets or no alphabets means that they were not significantly different. A significant difference was observed at *p* < 0.05.

### 3.3 Sequence description analysis

**Fig. 1:**
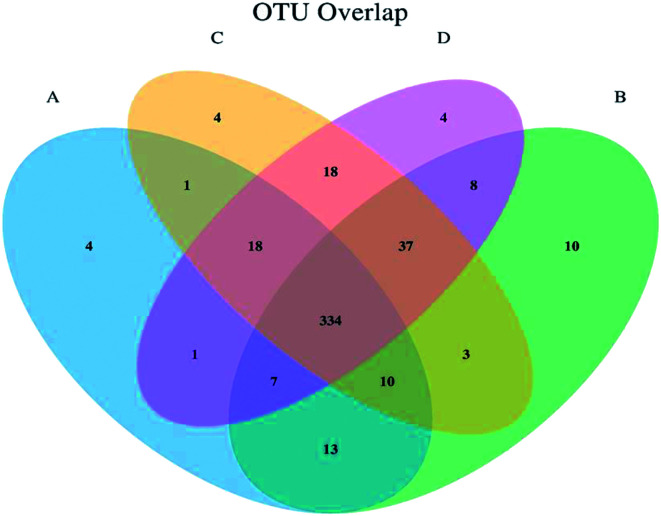
Venn diagram showing the overlap between groups. The Venn diagram represents the OTUs similarities and dissimilarities shared between samples. The letters A, B, C, and D represent the treatment groups, and the numbers represent the OTUs.

The high throughput pyrosequencing of the cecal samples produced 65 590 overlapping pair-end reads from the 16 samples; 61 618 clean reads were obtained representing 93.94% of reads to be subjected to further analysis. The clean reads were combined to tag bases. About 30 809 total reads were obtained with a mean of 460.32 tags per sample, and all reads were assembled. 27 867 normalized tags of the OTUs were obtained with total aligned tags of 25 094. The aligned rate per sample was 90.05%, and a total number of OTUs of 210 was obtained by clustering the 30 809 tags at 97% similarity (Table 4). The overlap of OTUs between groups was represented in a Venn diagram (Fig. 1). The Venn diagram estimates the overlapping OTUs composition and species overlap between the treatment groups. Fig. 1 showed that 334 OTUs were shared among all sample groups. Higher species specificity was observed in treatment B with 10 OTUs, while treatment A, C, and D have OTUs specificity of 4 each. The shared OTUs between the groups were 37 and between treatments B, C, and D; 7 OTUs were shared between treatments A, B, and D; 10 OTUs were shared between treatments A, B, and C. 18 OTUs were shared between A, C, and D. The shared OTUs between two sample treatments were 18 between treatment C and D, 1 OTU between treatment A and C, and 1 OTU between treatment A and D, 3 OTUs between B and C, and 8 OTUs between B and D (Fig. 1).

**Table 4:** Data sequencing statistical analysis involving data filtering statistics, sequencing splicing, and OTU statistics^a^

Sample	Statistics
**Filtering statistical**	
Raw reads	65 590
Raw bases (bp)	16 397 500
Clean reads	61 618
Clean reads rate (%)	93.94
Clean bases (bp)	18 177 310
Low-quality reads	3972
Low-quality reads rate (%)	6.06
Ns reads	0.0
Ns reads rate(%)	0.0
Adapter polluted reads	0.0
Adapter polluted reads rate (%)	0.0
Raw Q30 bases rate (%)	80.63
Clean Q30 bases rate (%)	82.32

**Sequence splicing**	
Total reads	30 809
Total assembled reads	30 809
Total assembled rate (%)	100.0
Average assembled length	460.32
Std assembled length	9.39

**OTUs summary statistics**	
Normalized tags	27 867
Total aligned tags	25 094
Aligned rate (%)	90.05
Total OTUs number	210

a Notes: Table 3 represents the data sequencing analysis involving data filtering statistics, sequencing splicing, and OTUs statistics.

### 3.4 Gut microbiota richness and diversity

**Fig. 2:**
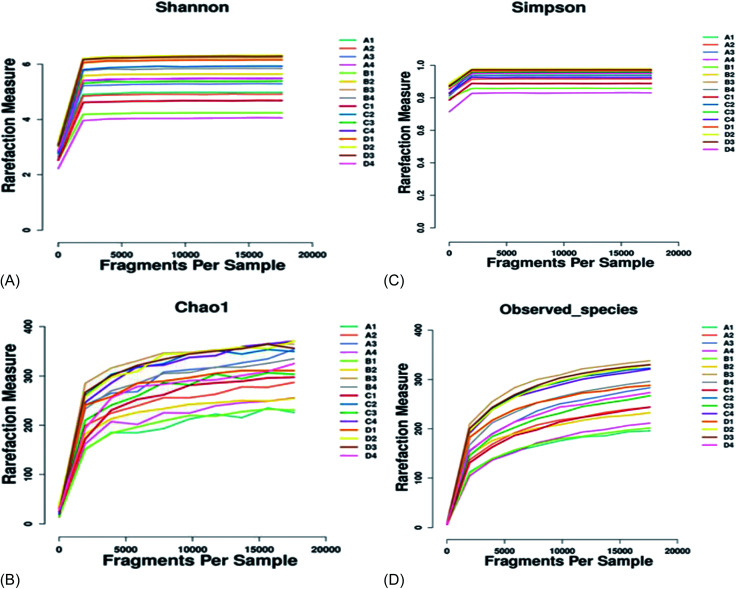
The alpha indices estimating species richness and diversity between groups. (A) The Shannon diversity index, (B) the Chao 1 index, (C) the Simpson diversity index, and (D) Observed_species.

**Fig. 3:**
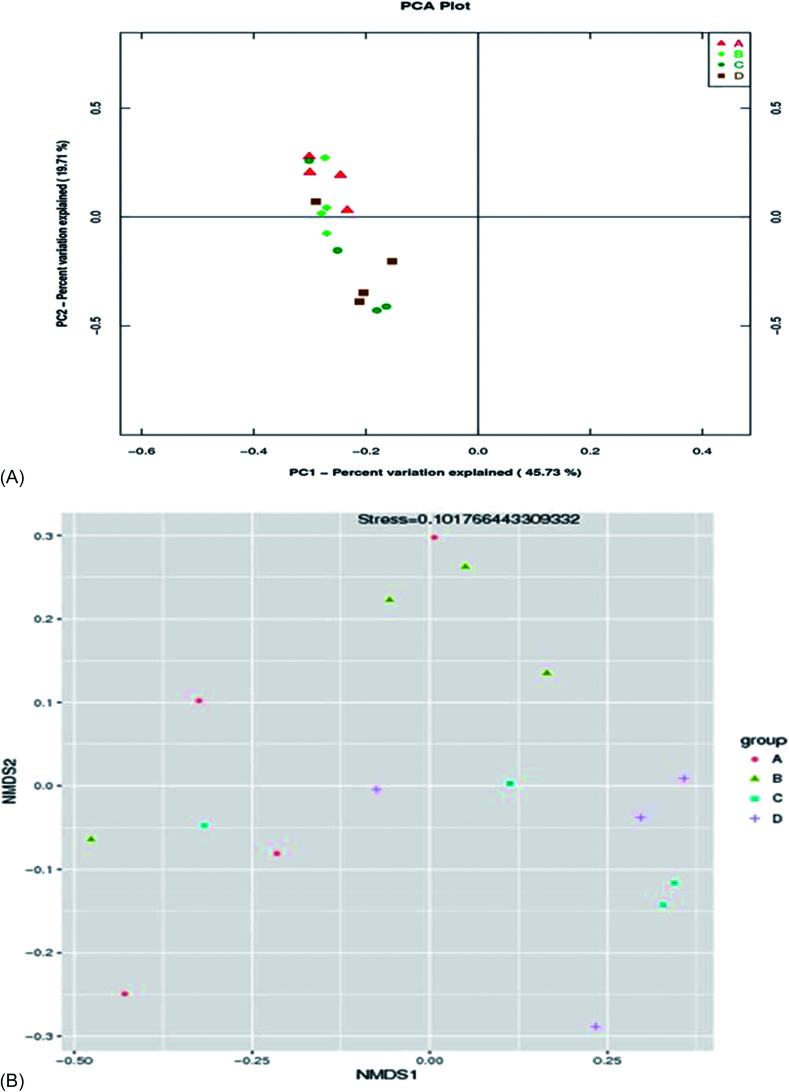
The PCA (A) and NMDS (B) of species similarity and dissimilarity between groups.

There was no significant difference (*p* > 0.05) in species richness, as represented by the Chao 1 and Observed_species values in the treatments (Table 5 and Fig. 2). The species richness increased with the increase in the supplementation of AF, reaching a plateau and then decreasing as the AF content increased (treatment D). The highest Chao 1 and Observed_species values were recorded in treatment B and the lowest in the control group (A). There was no significant difference (*p* > 0.05) in species diversity in all treatment groups. The highest Shannon diversity index value was found in treatment B, and the lowest Shannon diversity index was observed in treatment D. The Simpson diversity value increased with the increasing AF content. The highest Simpson diversity index was observed in treatment D and the lowest in treatment A. The PCA and non-metric multi-dimensional scale method (NMDS) reflects the differences between samples in the two-dimensional coordinate map (Fig. 3B). The more similar the composition of the microflora between the samples, the closer the distance representing their coordinate points in the PCA plot. The contribution rates of PC1 and PC2 in Fig. 3A were 45.73% and 19.71%, respectively, with a cumulative contribution rate of 65.44%. On the PC2 axis, treatments A and B were distributed towards the positive direction, while treatments C and D were mainly distributed towards the negative direction. The supplementation of AF increased the microbial community distribution in each sample, but the microbial composition between the treatments was similar. The closer the PCA plot in the distribution principal components in Fig. 3A, the more similar the community composition of the samples.

**Table 5:** Effects of AF on microbial species richness and diversity^a^

Items	A	B	C	D
**Richness index**				
Chao 1	312.39 ± 36.59	318.23 ± 56.84	316.77 ± 28.87	311.29 ± 5.00
Observed_species	246.1 ± 35.91	288.77 ± 53.18	277.3 ± 39.57	263.67 ± 30.20

**Diversity index**				
Shannon	5.18 ± 0.12	5.91 ± 0.30	5.33 ± 0.62	5.18 ± 0.11
Simpson	0.89 ± 0.06	0.93 ± 0.05	0.92 ± 0.03	0.96 ± 0.01

a Notes: Table 4 represents the effects of AF on microbial richness and diversity. The microbial richness was represented with the Chao 1 index and the Observed_species index, and the microbial diversity was represented with the Shannon and Simpson diversity indices. The letters A, B, C and D represent the treatment groups. There was no significant difference (*p* > 0.05) in the microbial richness and diversity.

### 3.5 Cecal microbiota composition

**Fig. 4:**
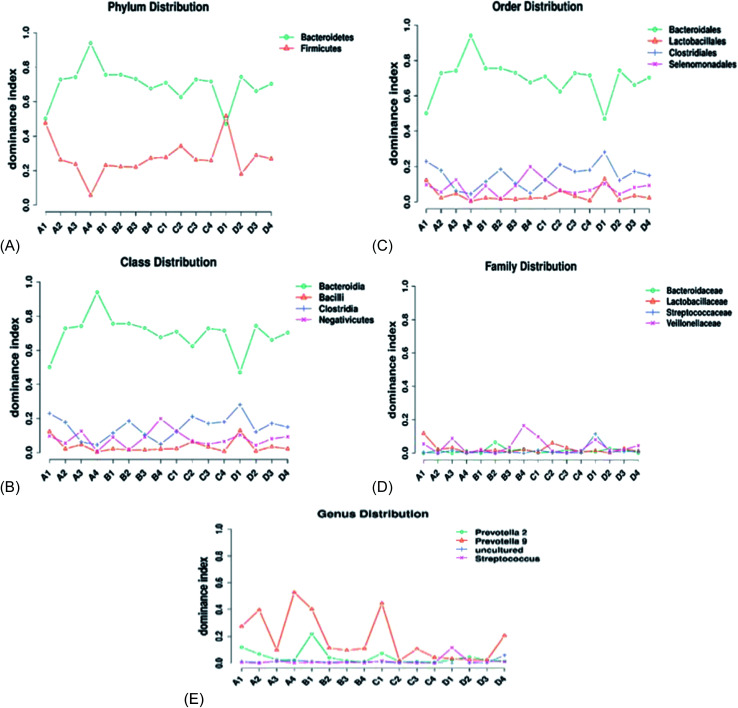
Line graph representing the distribution of the dominant bacteria species in the taxa. (A) Phylum distribution, (B) class distribution, (C) order distribution, (D) family distribution, (E) genus distribution.

The effects of AF on piglets cecal microbial composition were observed at different taxonomic levels. With regard to the phyla, a total of 15 phyla were identified in each sample. There was no significant difference (*p* > 0.05) in the phylum microbial composition between the treatment groups and the control. *Bacteroidetes* and *Firmicutes* were the main dominant phyla, accounting for about 98% of all reads (Fig. 4A). The *Bacteroidetes* phylum was significantly higher (*p* < 0.05) in all treatment groups compared with the other bacterial phyla. The *Bacteroidetes* was the most dominant phylum; the relative abundance of the *Bacteroidetes* decreased slightly upon the addition of AF and then increased at a steady rate as the AF concentration increased. The *Firmicutes* concentration increased with the increasing AF concentration; at the highest AF level, there was a decreasing trend observed (Fig. 4A).

At the class level, a total of 23 bacterial classes were observed across all treatment groups (Fig. 4B). Our results indicated that *Bacteroidia*, *Bacilli, Clostridia*, and *Negativicutes* were the dominant bacteria classes. The relative abundance of *Bacteroidia* was significantly different (*p* < 0.05) in all treated groups compared to the other bacteria classes. The *Bacilli* significantly increased (*p* < 0.05) in treatment C compared to the other treated groups. The *Negativicutes* class was not significantly affected by the addition of AF in the diet. According to the increasing order of the dominant bacteria classes, *Bacteroidia* > *Bacilli* > *Clostridia* > *Negativicutes*, accounting for 95% of the total reads (Fig. 4B).

A total of 26 bacteria orders were identified in each treatment (Fig. 4C). The cecal microbial composition was dominated by the following orders: *Bacteroidales* (65%), *Lactobacillales* (15%), *Clostridiales* (10%), and *Selenomonadales* (5%) (Fig. 4C). The *Bacteroidales* was significantly different (*p* < 0.05) in all the treatment. The *Bacteroidales* decreased as the concentration of AF increased. The *Clostridiales* increased with an increase in AF levels. The *Selenomonadales* order increased with the increase in AF supplementation. The *Lactobacillales* levels maintained a steady state level with the increase in AF; at the highest AF level (D), there was a reduction in the relative abundance of *Lactobacillales*.

At the family level, 26 families were characterized within each treatment group (Fig. 4D). *Bacteroidaceae*, *Lactobacillaceae*, *Streptococcaceae*, and *Veillonellaceae* were the dominant bacteria families. The supplementation of AF had no significant effect on the *Bacteroidaceae* and *Streptococcaceae* families in each treatment group. There was a slight increase in the relative abundance of *Lactobacillaceae* and *Veillonellaceae* as the AF content increased (Fig. 4D).

At the genera level, 26 genera were characterized (Fig. 4E). *Prevotella 2*, *Prevotella 9*, uncultured, and *Streptococcus* were the dominant species in all treatments (Fig. 4E). The relative abundance of *Prevotella 9* was significantly dominant (*p* < 0.05) in all treatment groups compared with the other bacterial genera. The supplementation of AF in the diet of piglets slightly increased the relative abundance of *Prevotella 2* and *Prevotella 9* at higher concentrations of AF (D). AF treatment has no effect on the Uncultured and the *Streptococcus* bacterial populations.

### 3.6 Cecal microbial network

**Fig. 5:**
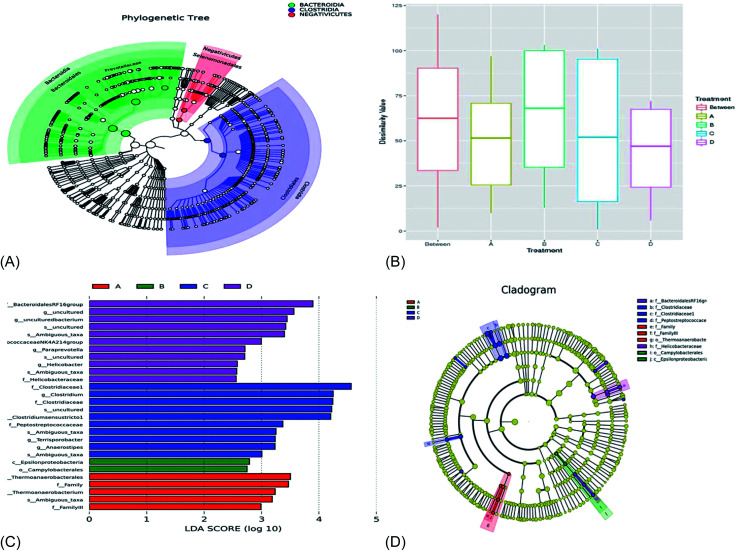
(A) Species evolution tree, (B) anosim analysis of taxa similarity, (C) LDA value distribution histogram example of the LefSe evolution branch diagram; (D) the cladogram of LEfSe demonstrates the taxonomic profiling for the distinct bacteria with significant higher abundances. The circles from inwards to outwards represent the different levels of bacteria members ranged from phylum to genus. The results were analyzed by the wilcoxon rank-sum test and are presented as the mean relative abundance with one asterisk meaning *p* < 0.05. LEfSe, linear discriminant analysis effect size.

Fig. 5A showed that the bacterial members with significantly high abundance mainly belonged to the *Bacteroidia*, *Clostridia*, and *Negativicutes* classes. The bacterial taxa with significantly high population abundance were dominated by the *Bacteroidales*, *Clostridiales*, and *Selenomonadales* orders in the treatment groups. The bacterial taxa with significantly greater population were dominated by the *Prevotellaceae* family (Fig. 5A). From Fig. 5B, the *R*-value was −0.083 and the *p*-value was 0.662, implying that the *R*-value was closer to 0 and the *p*-value was greater than 0.05. This means that there was no significant difference (*p* > 0.05) between and within the treatment groups.

From Fig. 5C, 11 bacterial taxa were shown to be specific to treatment D and, hence, influence the community structure. A total of 10 bacterial taxa were specific to treatment C; these bacterial taxa were specific and influenced the community structural composition in treatment C. 2 bacterial taxa were specific and influenced the community structural composition in treatment B. In treatment A, 5 bacterial taxa were specific and influenced the bacterial composition. Fig. 5D showed that the bacterial members with significantly high abundance mainly belonged to the *f_Bacteroidales RF16* group and *f_Helicobacteraceae* families in treatment D. In addition, the bacterial taxa with significantly greater population were dominated by the *f_family*, *f_family III*, and *O_campylobacterales* order in treatment A. In treatment B, the relative abundance of the bacterial taxa with significantly greater population were the *O_campylobacterales* and *C_Epsilonproteobacteria* classes. There were three families with significantly dominant bacteria population, *f_Clostridiaceae*, *f_Clostridiaceae 1*, and *f_Peptostreptococceae*, in treatment group C.

## 4 Discussion

Early weaning is a normal husbandry practice in most global swine production.^46^ Weaning is a harsh condition that is mostly associated with growth disorders and health effects in piglets. Piglets often exhibit a reduction in feed consumption and low nutrient metabolism due to their immature digestive systems, stress as a result of the separation from the dam and littermates, and the sudden change from the easily digestible sow's milk to a less digestible solid feed.^26^ These piglets have limited susceptibility to diseases and infections from enteric bacteria in addition to frequent diarrhea, stunted growth, low nutrient utilization efficiency, and even mortality.^31,47^ Due to the reduction in small intestinal composition, limited immunological response, and other protections derived from the maternal milk, piglets are more susceptible to diseases and infections by enteric bacteria like *E. coli* and *Salmonella*.^46,47^ There have been reports that the adaptation of piglets to a weaning diet takes more than 14 days.^48^ The composition and functions of the gut microbiota can be influenced by dietary nutrient supplementation.^49^ Therefore, the objective of this study was to investigate the prebiotic effects of AF supplementation to the diet of early weaning pigs on cecum microbial composition and function, piglet diarrhea, and SCFAs production.

In global pig farming, several dietary-fiber-based feeds have been used to increase the nutrient digestibility and growth performance of pigs^50^ and improve their gut microbial composition. However, the effects of dietary fiber supplementation on growth performance in pigs are inconsistent.^36^ Many studies have indicated that high dietary fiber supplementation increased growth,^51–53^ while others observed no impact on growth performance.^29,30,54^ The changes in the gut milieus and nutrient digestion can improve the gut microbial composition, production performance, and health of pigs. Previous studies in our group^28,46^ reported that *Astragalus membranaceus* fiber supplemented in a corn-soybean-based diet significantly increased the growth performance of piglets. Similarly, the findings of this study showed that the supplementation of AF increased the growth performance of piglets, but at higher AF concentrations, there was a decrease in growth performance. This result implies that increasing the AF content of the diet may improve the gut milieus, increase growth, and consequently improve the gut microbial composition of piglets, but at higher AF levels, there may be a detrimental effect on the growth performance of piglets.^26^ The findings of this current study were consistent with our previous investigation^31,46^ and that of other reports.^51,55^ The reason for the observed decreased in growth performance of piglets at high AF levels may be that piglets cannot metabolize high-fiber diets due to their immature digestive tracts and the low microbial composition.^26^ In contrast to our findings,^56,57^ no effects were reported on the ADFI, ADG, or FCR after supplementing dietary corn bran to the diet of weaned piglets. Our results further elucidated that the supplementation of AF to the diet of piglets significantly decreased diarrhea incidence in comparison with the control. This observation was consistent with the results.^3,16,31,32,58^ This observation may be due to the rich bioactive compound composition of AF, which may exert anti-inflammatory and anti-microbial effects on the gut of weaning piglets thereby reducing the population of enteric bacteria in the gut. Therefore, decreasing the diarrhea incidence in pigs.

We further evaluated the effects of AF on SCFAs production in pigs. Short-chain fatty acids, also known as volatile fatty acids (VFA), mainly include acetic acid, propionic acid, and butyric acid, which are involved in organ metabolic processes in the body and exert their respective functions. The synthesized acetic acid enters the blood and participates in the metabolic processes of the liver, heart, spleen, and the brain.^59^ Propionic acid and butyric acid not only provide energy for liver metabolism but also inhibit cholesterol synthesis; these SCFAs regulate the expression of genes and maintain stability within the intestinal tract. From the results of this experiment, the contents of acetate, propionate, and butyrate in the cecum of the AF-fed groups were higher than that of the control group. This study was consistent with the findings.^15,31^ Also, Gidenne *et al.* found that an increase in fiber levels can increase the concentration of VFAs in the cecum and concluded that a high-fiber diet can be fermented in the cecum to produce SCFAs to provide energy for the body.^60^ However, acetate and propionate content decreased at higher dietary AF levels, indicating that excess fiber may decrease the microbial activities in the cecum. The butyrate levels significantly increased with the increase in dietary AF levels. In contrast to our findings, Zhang *et al.* reported lower ileal VFA concentrations in pigs fed corn-based diets.^61^ In addition, Freire *et al.* reported low VFA production in pigs after changing the diet from sugar-beet pulp to soybean hulls.^62^ Conversely, insoluble dietary fiber can decrease the transit time, leading to a reduction in nutrient absorption in the small intestine.^52,56^ Short-chain fatty acids such as propionic and butyric acids were reported to activate the GPR43 on entero-endocrine l-cells to increase gut hormonal synthesis, such as PYY and GLP1, which play vital roles in appetite regulation through the gut–brain axis.^56^ High fiber intake increased the synthesis of PYY and GLP in addition to inhibiting energy consumption and increase satiety.^56,63^ Therefore, the increase in the synthesis of appetite-stimulating hormones through the consumption of dietary fiber coupled with the nutrient-rich AF diet may be a vital insight into the observed increase in the growth performance and synthesis of energy metabolites. Moreover, the mechanism of action may be due to the positive impact of AF on the cecal microbial composition of piglets.

The gut contains large volumes of microorganisms that play vital roles in both human and animal well-being. High throughput 16S-rDNA sequencing was used in this study to evaluate the abundance and diversity of the cecum microbial composition in piglets. The supplementation of AF regulates the cecum microbial richness and diversity by enhancing the production of SCFAs through the fermentation of fibers. The knowledge of the diversity of the gut microbial composition during the production period in animals is very important.^64^ The microbial richness and diversity analysis indicates that there was no significant difference in species richness and diversity between the gut microbiota in all treatment groups. However, AF supplementation increased species richness in treatment B and decreased as the fiber content of the diet increased. The Shannon diversity first increased and later decreases as the AF content of the diet reached higher levels, while the Simpson diversity increased with the increase in AF levels. Our findings were consistent with the results of Che *et al.*, who observed an increase in species richness and diversity after incorporating *Astragalus membranaceus* fiber in the diet of piglets.^31^ In addition, Ran *et al.* reported an increase in the species richness and observed OTUs after supplementing thymol and carvacrol in the diet of hybrid tilapia.^65^ In contrast with our study was the findings of Liu *et al.*, who reported that there were no differences in the species diversity and richness in the colonic microbial composition of pigs.^56^ In the cecal gut microbiota of piglets, *Bacteroidetes* (72%) and *Firmicutes* (26%) were the dominant bacterial phyla. Consistent with our findings was the study by Lamendella *et al.*, who reported that *Bacteroidetes* and *Firmicutes* were the dominant bacteria in the pig cecum.^66^ Similarly, other studies^31,33^ reported that the *Bacteroidetes* and *Firmicutes* were the dominant bacterial phyla in the cecum of piglets. In addition, recent researchers reported that *Firmicutes* and *Bacteroidetes* were the predominant phyla in the gut microbiota of pigs.^33,56,67–69^ We observed that the relative abundance of *Bacteroidetes* was higher than the relative abundance of *Firmicutes*. Similarly, Klein-Jöbstl *et al.* reported that *Bacteroidetes* was the highest relative abundant phylum in the gut of calves.^64^ In contrast, Mateos *et al.* and Kim *et al.* reported that *Firmicutes* was the highest relatively abundant phylum in cattle.^70,71^ The supplementation of AF in the diet increased upon increasing the relative abundance of *Firmicutes*, while the *Bacteroidetes* population decreased upon the addition of AF. The changes in these two microbial strains may affect the metabolism and functions of the gut microbiota. Similarly, Li *et al.* reported that the addition of essential oils increased the *Firmicutes* phylum population while decreasing the relative abundance of the *Bacteroidetes* phylum.^3^ In contrast to this observation, Liu. *et al.* reported a decrease in the *Firmicutes* phylum population and an increase in the *Bacteroidetes* phylum after feeding a corn-based diet to piglets.^56^ The reason for the high abundances of *Bacteroidetes* was reported in previous studies^72,73^ that the *Bacteroidetes* were the main degraders of complex polysaccharides in the gut of pigs due to their ability to encode genes for glycoside hydrolases such as β-xylosidases, endo-1,4-β-xylanases, α-*N*-arabinofuranosidases, and polysaccharide lyases compared with the *Firmicutes* phylum or other bacterial phyla in the gastrointestinal tract. Meanwhile, recent studies have indicated that the *Firmicutes* to *Bacteroidetes* ratio is of significance in human gut microbial composition and functions in regards to energy metabolism and health.^74^ For example, Turnbaugh *et al.* reported that a higher *Firmicutes* to *Bacteroidetes* ratio improved the digestibility of nutrients in pigs.^75^ Related studies indicated that the *Bacteroidetes* in the cecum can encode glycosidic hydrolysis and polysaccharide cleavage genes that promote polysaccharide degradation,^76^ while *Firmicutes* play a role in digesting carbohydrates,^72^ which can extract more energy through the fermentation of fiber to synthesize more SCFAs. The relative abundance of the *Firmicutes* in groups B and group C was slightly higher than that in the control group. Consistently, this indicating that dietary fiber can promote the growth of the *Firmicutes* phylum.

## 5 Conclusions

In summary, piglets were fed with different concentrations of dietary AF for 48 days. The prebiotic effect of dietary AF was investigated on the diarrhea incidence, SCFAs production, and microbiota composition in weaning piglets. Dietary inclusion of AF in the diet of weaning piglets increased their growth performance and decreased diarrhea occurrences. In addition, the added AF content promoted the composition and diversity of the gut microbiota of piglets. However, the functions of the different microbiota inhabiting in the cecum of piglets were not estimated. Therefore, further research is recommended to elucidate the functional roles of all the microbial species involved in growth promotion, SCFAs synthesis, nutrient digestion, immunity, and energy metabolism.

## Conflicts of interest

The authors declare no conflicts of interest.

## Supplementary Material
